# *Aprica*: a new genus and life history for the pteridivore *Xanthiapatula* Druce, 1898 (Lepidoptera, Noctuidae)

**DOI:** 10.3897/zookeys.866.27647

**Published:** 2019-07-24

**Authors:** Paul Z. Goldstein, Daniel H. Janzen, Winnie Hallwachs

**Affiliations:** 1 Systematic Entomology Laboratory, USDA, National Museum of Natural History, E-502, P.O. Box 37012, MRC 168, Washington, DC 20013-7012, USA National Museum of Natural History Washington United States of America; 2 Department of Biology, University of Pennsylvania, Philadelphia, PA 19104, USA University of Pennsylvania Philadelphia United States of America

**Keywords:** Area de Conservacion Guanacaste (ACG), Costa Rica, fern-feeder, pteridivory

## Abstract

*Aprica* Goldstein, **gen. nov.** is described to accommodate *Xanthiapatula* Druce, 1898. Recent discovery of its larva, which has been recorded eating foliage of species in six families of leptosporangiate ferns, suggest a possible subfamily assignment within the Eriopinae, but this cannot be substantiated based on adult morphology. This species has no obvious close relatives either among the core noctuid pteridivore genera currently recognized in the Eriopinae (e.g., *Callopistria* Hübner, [1821]), nor among genera more recently discovered to be fern-feeders but which remain incertae sedis with respect to subfamily (e.g., *Leucosigma* Druce, 1908, *Lophomyra* Schaus, 1911). The recorded foodplant profile is similar to that of another ambiguously placed Nearctic species *Fagitanalittera* (Guenée, 1852) (Noctuidae: Noctuinae: Xylenini, incertae sedis) with which it shares no obvious synapomorphies.

## Introduction

Druce (1898: 486, pl. 94, fig. 14) described *Xanthiapatula* (Noctuidae) from the holotype collected in the Santa Clara Valley, Costa Rica. The species has subsequently been collected in southern Mexico and Central America. *Xanthia* is otherwise considered a holarctic genus feeding primarily on *Salix* and *Populus* (Salicaceae). The species *patula* was placed in in *Bagisara* by Poole (1989). In his treatment of New World Bagisarinae, a subfamily with larvae associated primarily with Malvaceae, Ferguson (1997: 348) acknowledged that the species’ placement was not straightforward, and returned it to *Xanthia* by default, remarking “I could not place it to genus, but am sure that the species belongs somewhere in the large assemblage long known as the Amphipyrinae but now probably within the expanded concept of the Hadeninae of recent authors (e.g., Kitching & Rawlins [1998].” Kitching and Rawlins’ (1999[1998]) circumscriptions of both Amphipyrinae and Hadeninae have since been modified considerably, and for the purpose of providing context we note that they included Eriopinae, which are currently the taxonomic core of noctuids that feed on ferns (pteridivores) as a tribe within the Hadeninae. The combination *Bagisarapatula* reappeared only informally among determinations made by Poole in the course of a long-term caterpillar inventory of Area de Conservacion Guanacaste (ACG), northwestern Costa Rica (Janzen and Hallwachs 2016) where, importantly, it was discovered feeding on several species of ferns. Since pteridivory (fern-feeding) occurs in few noctuid groups and is unrecorded both for Bagisarinae and for the Xylenini, in which *Xanthia* resides, we wished to evaluate whether this species shares any of the primary features of the Eriopinae. The Eriopinae are not decisively circumscribed, but a combination of numerous adult and larval features have been articulated by Poole (1995), Fibiger and Lafontaine (2005), and Yen and Wu (2009).

## Materials and methods

Pinned specimens were examined with an incandescent light source. Genitalic preparations follow Clarke (1941) in part and Lafontaine (2004) but were stained with chlorazol black and slide-mounted in Euparal. Vesicae were everted prior to fixation. Dissections followed either an overnight room-temperature soak in supersaturated sodium hydroxide or a brief 15-minute heated soak and were examined with stereomicroscopes prior to slide-mounting. Wing preparations followed the procedure modified from Jaeger (2017): Following an overnight soak in a small stender dish with enough 50% EtOH solution to cover the wings and 10 drops of 6% NaCl, ethanol and bleach were added as necessary, depending on the ease with which scales could be cleared. Wings were stained overnight in Eosin Y. Photographs were made using the Microptics and Visionary Digital imaging systems and images manipulated with Adobe Photoshop (Adobe Systems, Mountain View, CA). Images of vesicae were taken in glycerin, held in a sectioned plexiglass cylinder affixed to a slide. Measurements were made with the aid of an ocular micrometer. Forewing (FW) length was measured from the center of the axillary area to the apex of the forewing. Terminology generally follows Forbes (1954) and Lafontaine (1998, 2004). The fine description of characters such as coloration, vesica, and immature stages is confined to the species description; the remainder are included in that of the genus.

### Repository abbreviations

The following abbreviations refer to collections from which specimens form the basis of this study:

**AMNH**American Museum of Natural History, NY, USA

**MNHUK** The Natural History Museum, London, UK

**USNM**National Museum of Natural History [formerly, United States National Museum], Washington, District of Columbia, USA

## Results

Although some of the larval characters that can be seen in *A.patula* are consistent with those in Eriopinae, including cephalic striping, oblique lateral striping and false eyespots on the first abdominal segment (Yen and Wu 2009), they are not diagnostic, and most of the larval characters that would corroborate the assignment of *Aprica* to Eriopinae (including setal characters and features of the spinneret) are not discernible from available images. Two features in the adults consistent with Eriopinae are the expression and configuration of M2 arising from the discal cell in the hind wing (Fig. 21) and non-uniform sclerotization of the phallus (Figs 24–27), which is primarily confined apically and ventrally (Yen and Wu 2009). Abdominal hair pencils and eversible coremata on the sacculus (Fibiger and Lafontaine 2005) typical of Eriopinae are lacking. *Aprica* also lacks the conspicuous genitalic and larval features that diagnose either Bagisarinae (Ferguson 1997) such as the fused valvae and the absence of the first two pairs of abdominal prolegs; nor can it be assigned to Xylenini or, thereby, *Xanthia* based on the characters summarized by Fibiger and Lafontaine (2005: 48).

### Systematics

#### Aprica

Taxon classificationAnimaliaLepidopteraNoctuidae

Goldstein gen. nov.

7d5ca55c-15e5-542d-bc73-a6deb9e27575

http://zoobank.org/796AEA59-E454-495E-9109-D1BD667D1863

##### Type species.

*Xanthiapatula* Druce, 1898.

##### Type locality.

Costa Rica.

##### Etymology.

*Aprica* (feminine) derives from the Latin *apricus*, sunny, open to the light.

##### Diagnosis.

*Aprica* may be diagnosed readily both from the appearance of the forewing and by the male and female genitalia. The bisection of the golden-orange FW and similar thoracic coloration from the sunset-reddish HW and similar abdominal coloration is distinctive. Although the male genitalia are unremarkable, the valve simple with a rudimentary, hook-like clasper, the combination of this feature with the absence of abdominal coremata, and the presence of M2 on the HW, differentiates *Aprica* from other genera with pteridivorous species in which either the clasper is absent and the coremata present (e.g., *Callopistria*, *Phuphena* Walker, 1858); from genera with the reverse condition (e.g., *Fagitana* Walker, 1865, in which coremata are absent but which bear a complex clasping apparatus and corresponding ridge on the female 8^th^ sternite), or in which the hindwing M2 is not expressed (e.g., *Leucosigma*, *Lophomyra*). In both *Apricapatula* and *Fagitanalittera*, the corpus bursae is elongate and the ductus seminalis arises from an appendix bursae located at the posterior (proximal) end of the corpus, a condition shared by several Eriopinae but usually uninformative at the generic level.

##### Description.

***Head.*** Antennae setose, biramous in males, uniramous in females; scaled above, cupreous. Labial palpi upturned, densely scaled. Eyes naked.

***Thorax.*** Thoracic vestiture golden orange, concolorous with forewing. *Wings*. General “background” coloration sharply bisected between forewing and hind wing, the former predominantly orange (as the thoracic vestiture) and the latter a reddish russett (as the abdominal vestiture); M2 faintly but clearly expressed on hindwing. *Legs*. One pair mid-tibial spurs, two pair on hind-tibiae; three rows of tibial spines on legs.

***Abdomen.*** Coremata absent; without brushes, pockets, or levers.

***Male genitalia.*** Uncus heavily setose; dorsal edges of tegumen straight, angled ventrally at roughly 45°, tegumen widest supra-medially; valvae medially situated, articulating with the vinculum in its dorsal half, setose throughout, of more or less constant width with a minor constriction at the cucullus; corona well developed; baso-costal processes of sacculus robust; clasper medially situated in valve, elongate with a sharply sclerotized apical hook at the cucullus; pleurite fused; juxta shield-shaped; transtilla well developed and paratergal sclerite evident, well fused; sacculus gently rounded.

***Female genitalia.*** Papillae anales flanged at postero-basal edge; posterior and anterior apophyses rod-like, not swollen apically, the anterior slightly shorter than the posterior. Antrum well developed; ductus seminalis arising from the appendix bursae, appendix bursae deriving dorsally from the posterior third of the ductus bursae; ductus bursae wide, elongate, tubular, with a 360° counter-clockwise torsion immediately posterior to the corpus bursae; corpus bursae, oblong, bearing a single transverse signum.

##### Immature stages.

Known from images of *A.patula*; see description below.

##### Distribution.

Mexico and Central America

#### Aprica patula

Taxon classificationAnimaliaLepidopteraNoctuidae

(Druce, 1898) comb. nov.

7d2965bd-d088-5235-a7ce-d163d87fea56

Figs 1–10 , 11–20 , 21 , 22, 23 , 24–27 , 28–29 , 30–37 , 38–45 , 46–53

Xanthia patula Druce, 1898 in Godman and Salvin 1898: 486, pl. 94, fig. 14; Hampson, 1908: 597, pl. 121, fig. 23; Ferguson, 1997: 348.
Bagisara patula : Poole, 1989: 154.

##### Holotype locality.

Costa Rica, Santa Clara Valley [BMNH].

##### Material examined.

38♂, 16♀ **COSTA RICA** (34♂, 15♀): The following label data precede individual unique voucher codes of the format yy-SRNP-xxxxxx (SRNP = Santa Rosa National Park, a unique identifier coined when the inventory was confined to Sector Santa Rosa of ACG in the early 1980’s) on all reared and light-trapped specimens from ACG (24♂, 9♀): Voucher: D.H. Janzen & W. Hallwachs DB: http://janzen.sas.upenn.edu Area de Conservacion Guanacaste, COSTA RICA.

All records of “on” a given plant species refer definitively to “feeding on.” Specimens lacking food plant records were light trapped in the forest and have a 6-digit suffix in their SRNP codes, while reared specimens have a 1–5-digit suffix.

**Alajuela Province**: Area de Conservacion Guanacaste (9♂, 4♀): *Males*: Sector Rincon Rain Forest: Estacion Caribe (melina), 10.8956, -85.29558, el. 391m: 11/09/2007, F. Quesada & R. Franco, collector, 07-SRNP-110152, USNMENT01463558; Sector Rincon Rain Forest: Estacion Caribe (melina), 10.8956, -85.29558, el. 391m: 11/10/2007, F. Quesada & R. Franco, collector, 07-SRNP-110402, USNMENT01463615; Sector Rincon Rain Forest: Jabalina, Manta Pizote, 10.97325, -85.31542, el. 288m: 09/30/2008, S. Rios & H. Cambronero, collector, 08-SRNP-107404, USNMENT01463664; Sector Rincon Rain Forest: Jacobo, 10.94076, -85.3177, el. 461m: larva on *Salpichlaenavolubilis*: 06/16/2014, ecl. 07/19/2014, Edwin Apu, collector, 14-SRNP-80751, USNMENT01463658; Sector Rincon Rain Forest: Manta Hugo, 10.8811, -85.2677, el. 491m: 03/14/2009, H. Cambronero & R. Franco, collector, 10-SRNP-107506, USNMENT01463699; Sector Rincon Rain Forest: Protrero Chaves, 10.93868, -85.32167, el. 433m: 8/19/2009, F. Quesada & H. Cambronero, collector, 09-SRNP-107666, Dissection 148312, USNMENT01441902; Quebrada Bambu, 10.9301, -85.25205, el. 109m: larva on *Lomariopsisvestita*: 09/18/2012, ecl. 10/13/2012, Cirilo Umaña, collector, 12-SRNP-76932, USNMENT01463565; Sector San Cristobal: Estacion San Gerardo, 10.88009, -85.38887, el. 575m: 04/29/2006, H. Cambronero & S. Rios, collector, 06-SRNP-103767, USNMENT01463650; Sendero Carmona, 10.87621, -85.38632, el. 670m: larva on *Thelypterisnicaraguensis*: 05/16/2005, ecl. 06/07/2005, Gloria Sihezar, collector, 05-SRNP-2726, USNMENT01463665. *Females*: Sector Rincon Rain Forest: Sendero Rincon, 10.8962, -85.27769, el. 430m: larva on *Salpichlaenavolubilis*: 03/23/2011, ecl. 04/23/2011, Jose Perez, collector, 11-SRNP-41357, USNMENT01463594; Sector Rincon Rain Forest: San Lucas, 10.91847, -85.30338, el. 320m: larva on *Thelypterisnicaraguensis*: 6/8/2011, ecl. 6/27/2011, Jorge Hernandez, collector, 11-SRNP-42773, Dissection 148174, USNMENT01463999; Quebrada Escondida, 10.89928, -85.27486, el. 420m: larva on *Thelypterisnicaraguensis*: 11/16/2010, ecl. 12/16/2010, Anabelle Cordoba, collector, 10-SRNP-44267, USNMENT01463798; Sector San Cristobal: Estacion San Gerardo, 10.88009, -85.38887, el. 575m: 04/30/2006, S. Rios & F. Quesada, collector, 06-SRNP-103899, USNMENT01463696. **Guanacaste Province**: Area de Conservacion Guanacaste (15♂, 7♀): *Males*: Sector Cacao: Cuesta Caimito, 10.8908, -85.47192, el. 640m: larva on *Pterisplumula*: 11/13/2007, ecl. 12/07/2007, Manuel Pereira, collector, 07-SRNP-47084, USNMENT01463600; Sector Cacao: Estacion Gongora, 10.88449, -85.47306, el. 557m: 09/12/2007, R. Franco & S. Rios, collector, 07-SRNP-111179, USNMENT01463585; Sector Cacao: Estacion Gongora, 10.88449, -85.47306, el. 557m: 09/12/2007, R. Franco & S. Rios, collector, 07-SRNP-111178, USNMENT01463617; Sector Cacao: Gongora Bananal, 10.88919, -85.47609, el. 600m: larva on *Pterisplumula*: 10/25/2005, ecl. 11/19/2005, Manuel Pereira, collector, 05-SRNP-48763, USNMENT01463599; Sector Cacao: Quebrada Otilio, 10.88996, -85.47966, el. 550m: larva on *Pterisplumula*: 09/17/2007, ecl. 10/11/2007, Manuel Pereira, collector, 07-SRNP-46183, USNMENT01463543; Sector Cacao: Quebrada Otilio, 10.88996, -85.47966, el. 550m: larva on *Pterisplumula*: 09/17/2007, ecl. 10/08/2007, Manuel Pereira, collector, 07-SRNP-46186, USNMENT01463693; Sector Cacao: Quebrada Otilio, 10.88996, -85.47966, el. 550m: larva on *Pterisplumula*: 09/17/2007, ecl. 10/07/2007, Dunia Garcia, collector, 07-SRNP-46181; Sector Cacao: Toma de Agua, 10.92956, -85.46512, el. 1160m: 08/09/2010, S. Rios & R. Franco, collector, 10-SRNP-112086, USNMENT01463590; Sector Pailas: Canopy Tours, 10.81262, -85.40248, el. 700m: 9/30/2016, H.Cambronero&R.Franco, collector, 16-SRNP-106142, USNMENT01464165; Sector Pailas: Canopy Tours, 10.81262, -85.40248, el. 700m: 06/11/2008, H. Cambronero & F. Quesada, collector, 08-SRNP-103411, USNMENT01463691; Sector Pitilla: Colocho, 11.0256, -85.41224, el. 390m: 03/19/2007, H. Cambronero & F. Quesada, collector, 07-SRNP-102458, USNMENT01463603; Sector Pitilla: Estacion Pitilla, 10.98931, -85.42581, el. 675m: 03/01/2006, S. Rios & R. Franco, collector, 06-SRNP-102362, USNMENT01463573; Sector Pitilla: Estacion Pitilla, 10.98931, -85.42581, el. 675m: 03/02/2006, R. Franco & F. Quesada, collector, 06-SRNP-102550, Dissection 148362, USNMENT01463556; Sector Pitilla: Estacion Quica, 10.99679, -85.39695, el. 487m: 08/29/2008, S. Rios & R. Franco, collector, 08-SRNP-105409, USNMENT01463651; Sector Pitilla: Estacion Quica, 10.99679, -85.39695, el. 487m: 08/29/2008, S. Rios & R. Franco, collector, 08-SRNP-105410, USNMENT01463621. *Females*: Sector Cacao: Quebrada Otilio, 10.88996, -85.47966, el. 550m: larva on *Pterisplumula*: 09/17/2007, ecl. 10/08/2007, Manuel Pereira, collector, 07-SRNP-46187, USNMENT01463536; Sector Cacao: Quebrada Otilio, 10.88996, -85.47966, el. 550m: larva on *Pterisplumula*: 9/17/2007, ecl. 10/8/2007, Manuel Pereira, collector, 07-SRNP-46182, USNMENT01463895; Sector Cacao: Quebrada Otilio, 10.88996, -85.47966, el. 550m: larva on *Pterisplumula*: 09/17/2007, ecl. 10/11/2007, Manuel Pereira, collector, 07-SRNP-46185; Sector Cacao: Quebrada Otilio, 10.88996, -85.47966, el. 550m: larva on *Pterisplumula*: 9/17/2007, ecl. 10/12/2007, Dunia Garcia, collector, 07-SRNP-46180, Dissection 148173, USNMENT01463897; Sector Cacao: Roca Verde, 10.89354, -85.43603, el. 835m: 08/12/2007, R. Franco & F. Quesada, collector, 07-SRNP-108036, USNMENT01463587; Sector Pitilla: Estacion Pitilla, 10.98931, -85.42581, el. 675m: 02/28/2006, S. Rios & H. Cambronero, collector, 06-SRNP-101627, USNMENT01463623; Sector Pitilla: Pasmompa, 11.02666, -85.41026, el. 400m: 07/31/2008, R. Franco & S. Rios, collector, 08-SRNP-104889, USNMENT01463671. **Other** (10♂,4♀): *Males*: COSTA RICA: Juan Vinas, Schaus & Barnes, coll., USNMENT01463893; COSTA RICA: Cartago, Orosi Estacion Tapanti Parque, 9 456’ N, -83 471’ W, 4062”, July 7–9 2008, 1275m, J. Bolling Sullivan, collector, Dissection 148364, USNMENT01463654; same data, USNMENT01463683; same data, USNMENT01463630; COSTA RICA: Cartago, Orosi Estacion, Tapanti Parque, LN-559900-194000, 1275 m, February 12–17, 2005, J. Bolling Sullivan, collector, USNMENT01463894; COSTA RICA: Cartago, Orosi Estaction, Tapani Parque, LN-559900-194000, 1275 m, February 12–17, 2005, J. Bolling Sullivan, collector, Dissection 148371, USNMENT01463577; same data, USNMENT01463681; COSTA RICA: Tuis, May 28–June 4, Schaus and Barnes, collectors, Collection WmSchaus, USNMENT01463564; COSTA RICA: Tuis, June, Schaus and Barnes, collectors, USNMENT01463648; COSTA RICA: San Jose 4000ft, Nov 06, Collection WmSchaus, USNMENT01463535. *Females*: COSTA RICA: Cartago, Orosi Estaction, Tapani Parque, LN-559900-194000, 1275 m, February 12–17, 2005, J. Bolling Sullivan, collector, USNMENT01463571; same data, USNMENT01463641; same data, USNMENT01463568; COSTA RICA: Carillo, Schaus and Barnes, collectors, USNMENT01463572. **GUATEMALA** (3♂,1♀): *Males*: GUATEMALA: Cayuga, May, Schaus and Barnes, collectors, USNMENT01463555; GUATEMALA: Cayuga, Sept, Schaus and Barnes, collectors, Dognin Collection, USNMENT01463529; GUATEMALA: Cayuga, Schaus and Barnes, collectors, Aug., Photo Noc.22, USNMENT01463672. *Female*: GUATEMALA: Retalhuleu, from L Thiel, S Sebastian, USNMENT01463544. **MEXICO** (1♂): Zacualpan [Veracruz] T21, USNM slide # 59037, USNMENT01463653.

**Figures 1–10.**
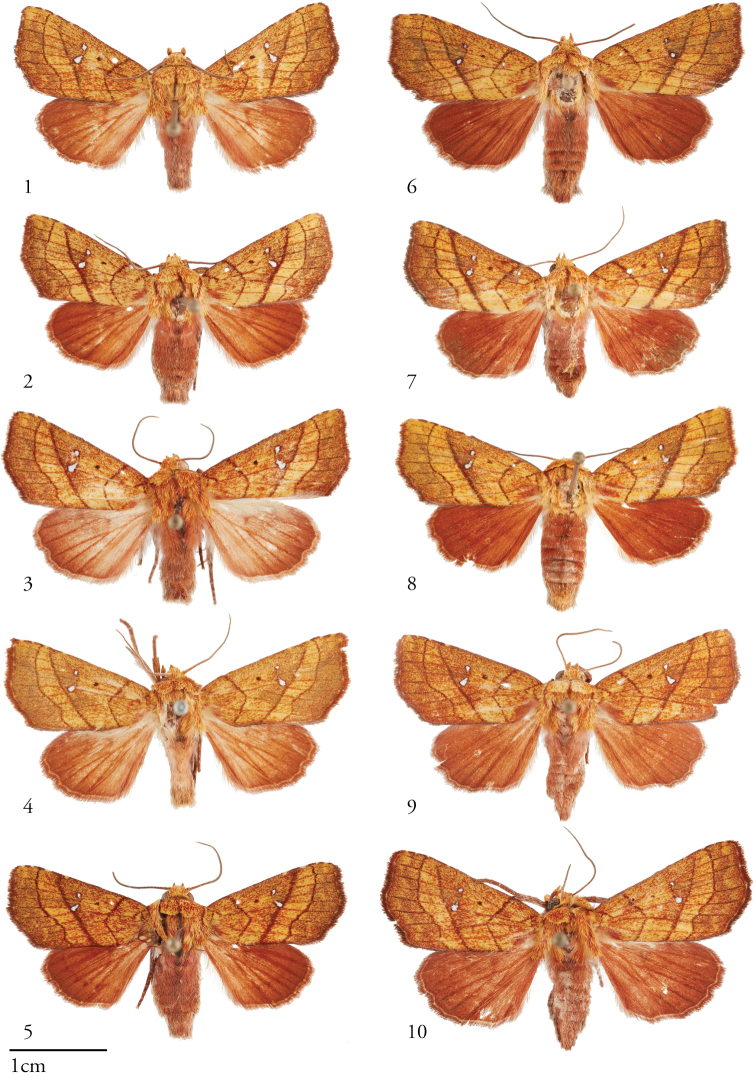
Dorsal habitus of *Apricapatula*, males (left, **1–5**) and females (right, **6–10**). **1**USNMENT01463599, 05-SRNP-48763 **2**USNMENT01463665, 05-SRNP-2726 **3**USNMENT01463894**4**USNMENT01463893**5**USNMENT01463658, 14-SRNP-80751 **6**USNMENT01463798, 10-SRNP-44267 **7**USNMENT01463999, 11-SRNP-42773 **8**USNMENT01463895, 07-SRNP-46182 **9**USNMENT01463671, 08-SRNP-104889 **10**USNMENT01463571.

**Figures 11–20.**
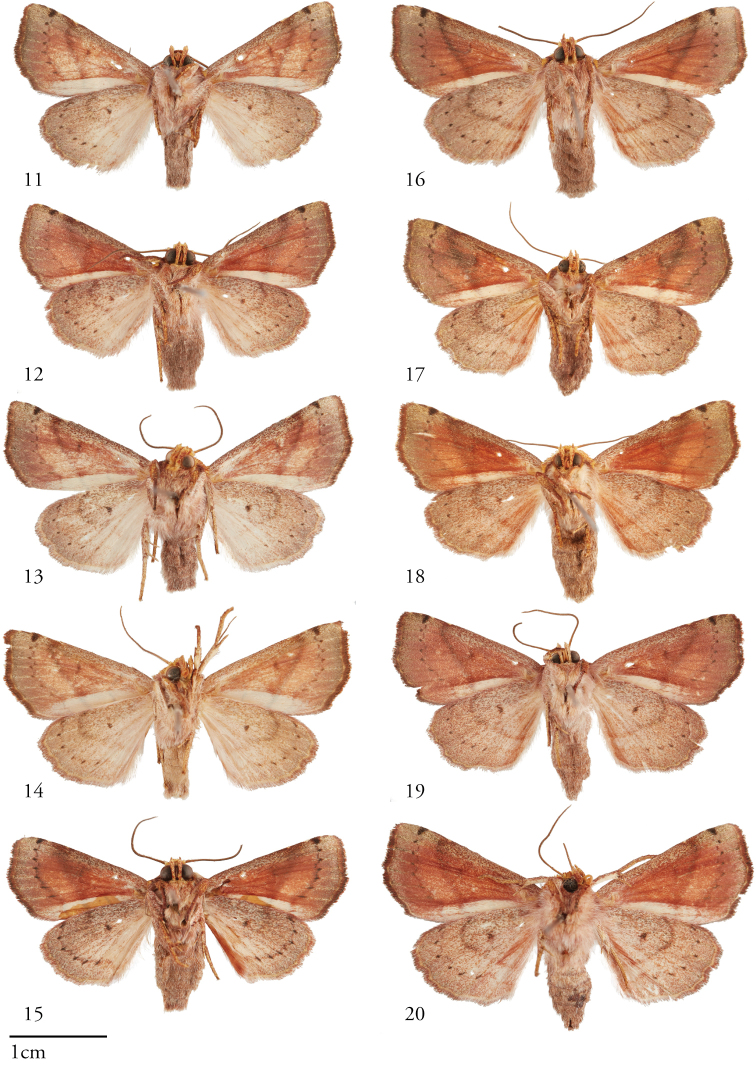
Ventral habitus of *Apricapatula*, males (left, **11–15**) and females (right, **16–20**). **11**USNMENT01463599 05-SRNP-48763 **12**USNMENT01463665 05-SRNP-2726 **13**USNMENT01463894**14**USNMENT01463893**15**USNMENT01463658 14-SRNP-80751 **16**USNMENT01463798 10-SRNP-44267 **17**USNMENT01463999 11-SRNP-42773 **18**USNMENT01463895 07-SRNP-46182 **19**USNMENT01463671 08-SRNP-104889 **20**USNMENT01463571.

**Figure 21.**
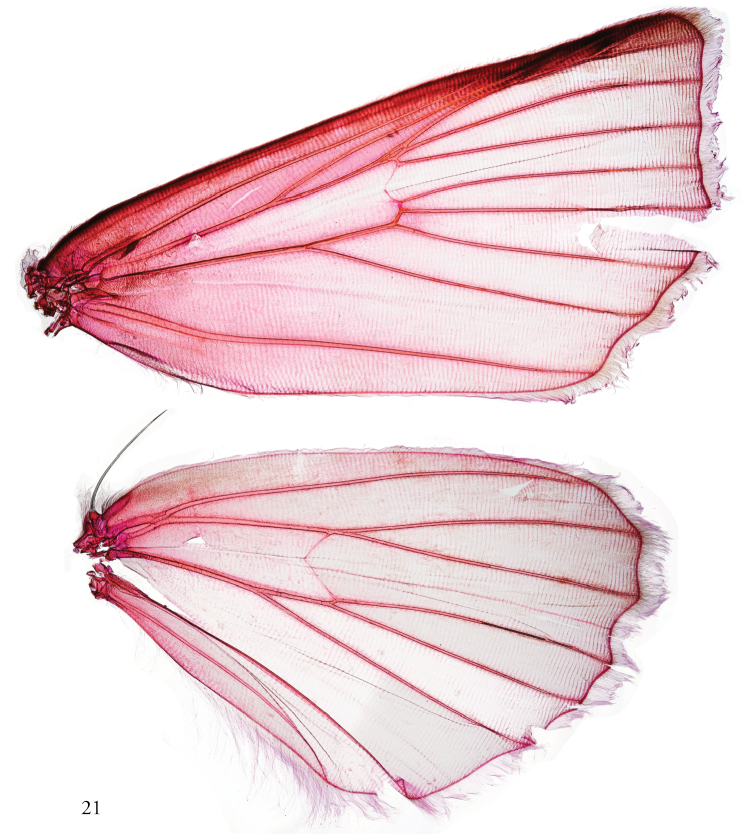
Forewing, hind wing. male, USNMENT01463577, Dissection #148371.

**Figures 22, 23.**
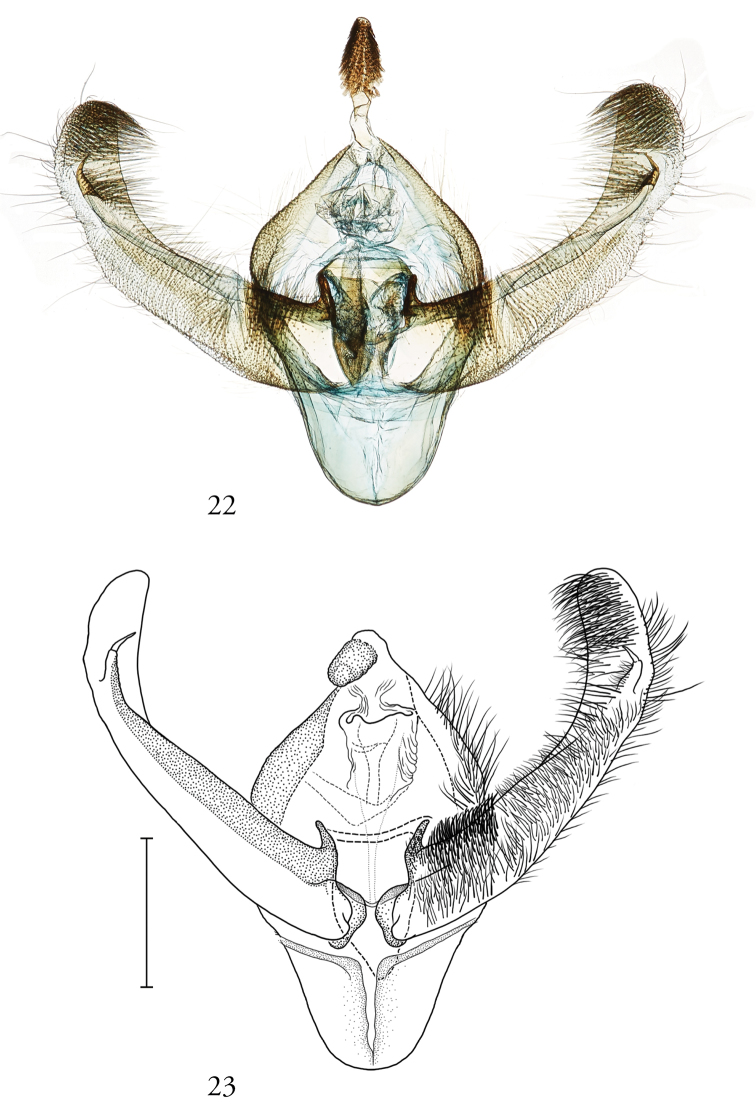
Male genitalia. **22** Valvae, USNMENT01463556, 06-SRNP-102550, Dissection #148362 **23** Valvae (ink) USNMENT01441902, 09-SRNP-107666, Dissection #148312.

##### Diagnosis.

The apposition of the forewing and hind wing colors differentiates this species from several unrelated New World species (both tropical and extra-tropical) that share superficially similar orange forewing coloration. None of these has a deep reddish hind wing or the laterally bisected contrast in body coloration between thorax and abdomen. The combination of the expressed M2 on the hind wing, the absence of abdominal coremata, and the configuration of the male and female genitalia are summarized in the generic diagnosis *supra vide* as distinct from Eriopinae and other pteridivorous Noctuidae.

##### Description.

***Head***. Eyes smooth; labial palpi upturned, apex level with antennal base; antennae setose, bifasciculate in males; frons and vertex mix of golden yellow and reddish-orange scales concolorous with those of forewings and thorax.

***Thorax***. Prothoracic vestiture as described for genus. *Wings*. Forewing length, males, 12.1 mm–15.0 mm (males, *n* = 34, *x̄* = 13.5 mm, M = 13.2 mm); females, 12.1 mm–16.1 mm (*n* = 17, *x̄* = 14.2 mm, M = 14.3 mm). FW not broadly rounded, outer margin convex; FW scaling golden yellow, suffused with reddish-orange scales, some lilacine at costa; postmedial area less heavily suffused with reddish-orange than antemedial or subterminal areas; antemedial, medial, and postmedial lines distinct and unbroken, the medial line ~2× as thick as others; baso-posterior russet patch; reniform spot constricted to form two white stigmata, the antero-costal stigma round and smaller than the other, j-shaped stigma; HW near-uniformly russet-orange, yellowish-orange terminal line unbroken. FW underside russet in center, bounded by paler shading along the costal and posterior margin below the anal vein; pm line jagged, dark gray, fading gradually from costal fascia to the anal vein. HW underside with discal spot present, pm line visible as a series of dark gray spots where it meets the veins; medial lines of both wings diffuse. *Legs*. As above, for genus. Scales the same mix of golden orange and reddish as on the head and thorax, but more uniformly reddish on the fore-femora.

***Abdomen***. As above, for genus. Vestiture paler than on thorax and concolorous with hind wing.

**Figures 24–27.**
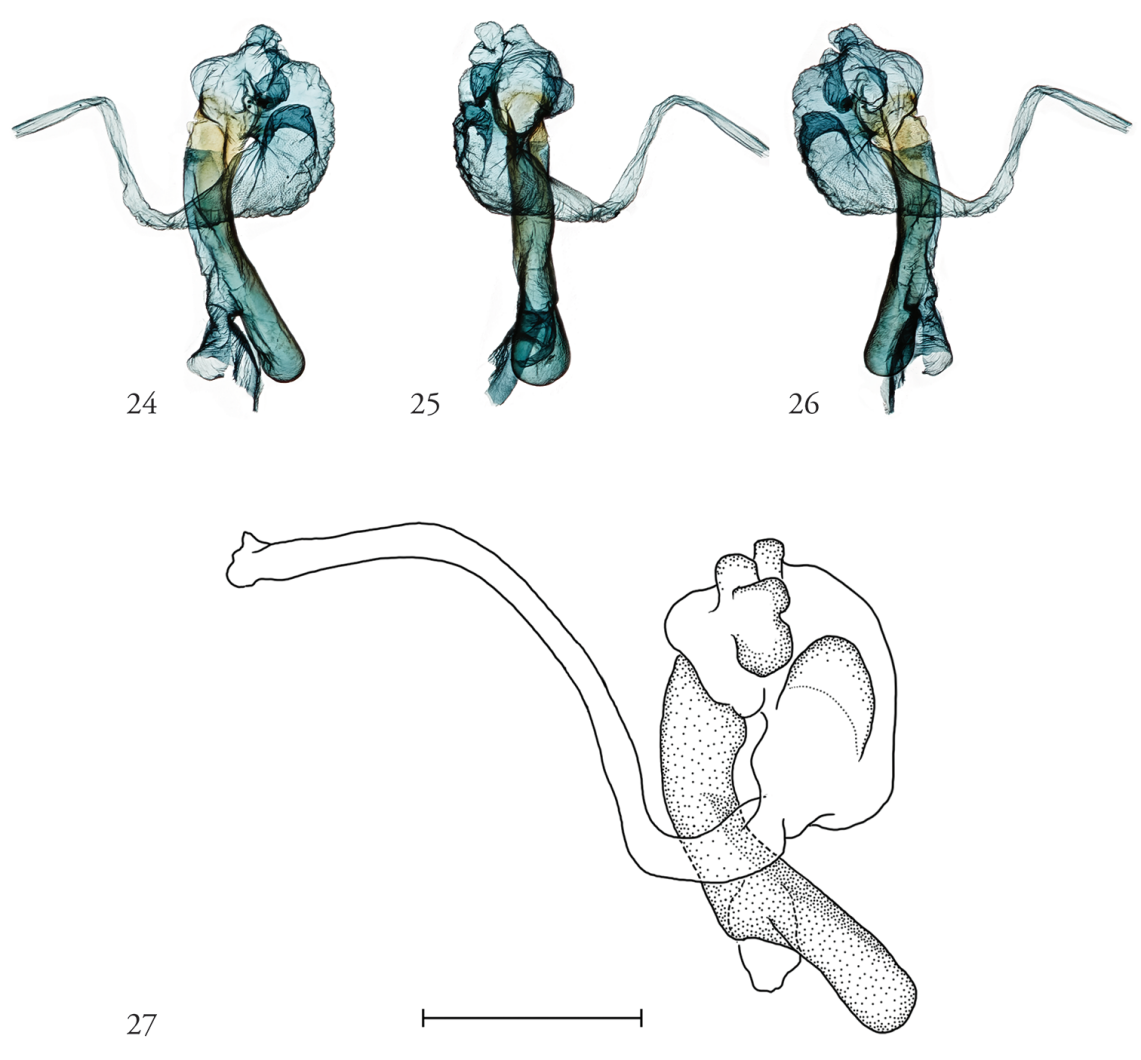
Male genitalia. **24–26** Vesica, from 3 angles, USNMENT01463556, 06-SRNP-102550, Dissection #148362 **27** Vesica (ink), USNMENT01441902, 09-SRNP-107666, Dissection #148312.

***Male genitalia***. As above, for genus. Phallus not uniformly sclerotized, weakly so towards the vesica; vesica without cornuti, with a complex of four bubble-like sub-basal diverticula and one larger basal diverticulum; vesica distended baso-medially, recurved clockwise over the phallus before narrowing and everting in a counter-clockwise twist (Figs 24–27).

**Figures 28–29.**
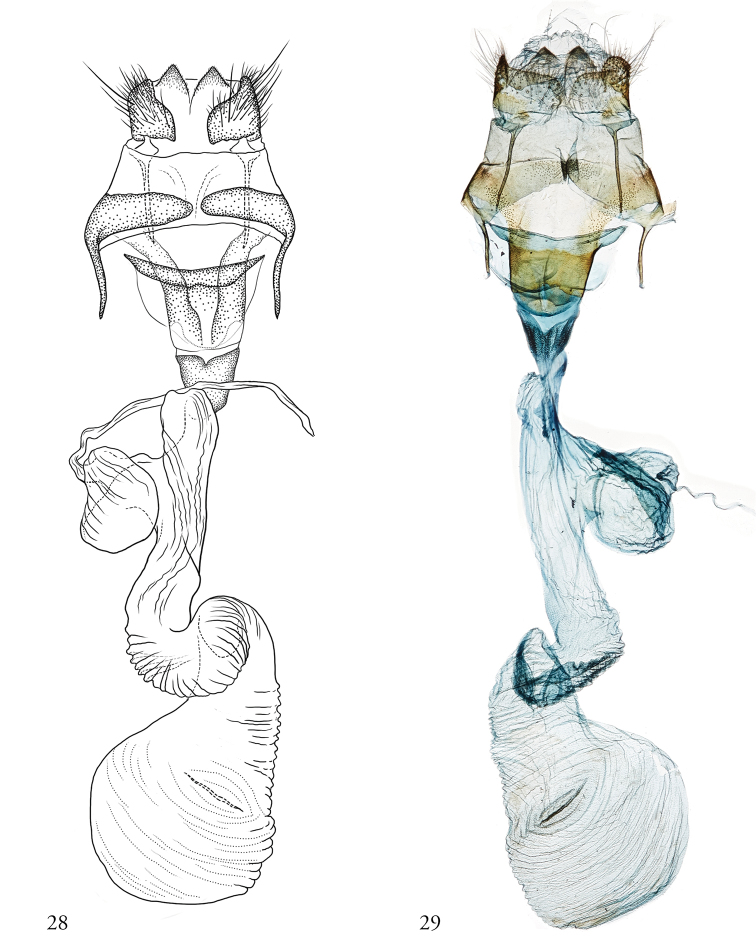
Female genitalia. **29**USNMENT01463999, 11-SRNP-42773, Dissection #148174 (ink) **30**USNMENT01463999, 10-SRNP-46180, Dissection #148173.

**Figures 30–37.**
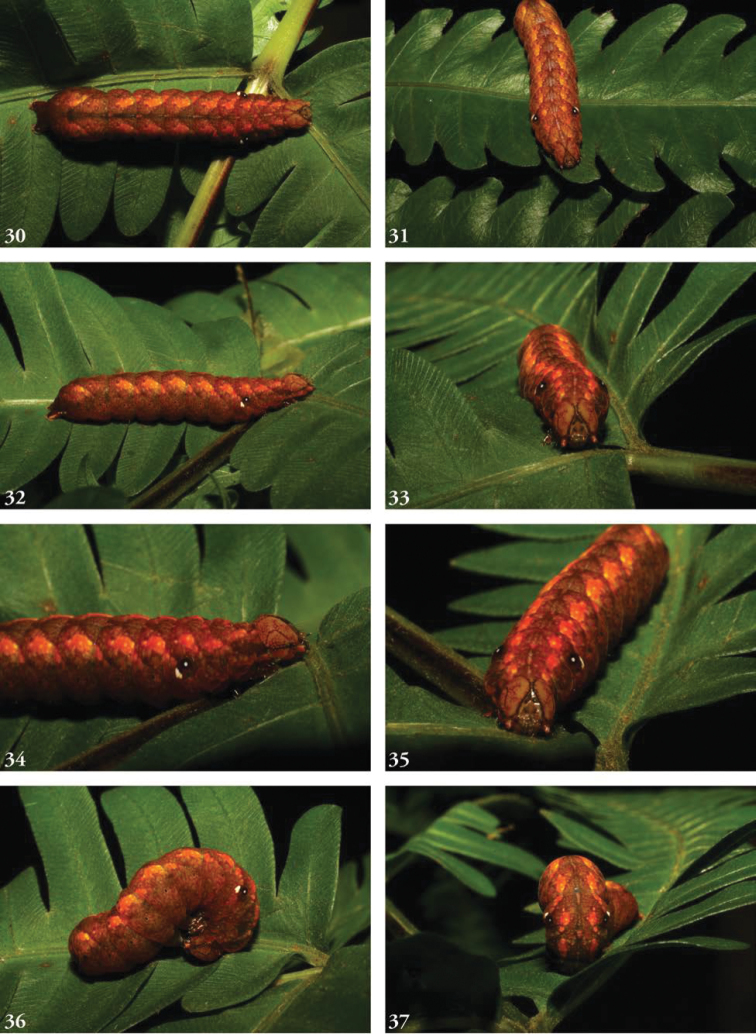
Larvae, brown, last instar. **31**USNMENT01463599, 05-SRNP-48763 ♂ DHJ408116 **32** SNMENT01463665 05-SRNP-2726 ♂ DHJ402483 **33**USNMENT01463599, 05-SRNP-48763 ♂ DHJ408117 **34**USNMENT01463599, 05-SRNP-48763 ♂ DHJ408121 **35**USNMENT01463599, 05-SRNP-48763 ♂ DHJ408123 **36**USNMENT01463599, 05-SRNP-48763 ♂ DHJ408124 **37**USNMENT01463599, 05-SRNP-48763 ♂ DHJ408125 **38**USNMENT01463599, 05-SRNP-48763 ♂ DHJ408126.

**Figures 38–45.**
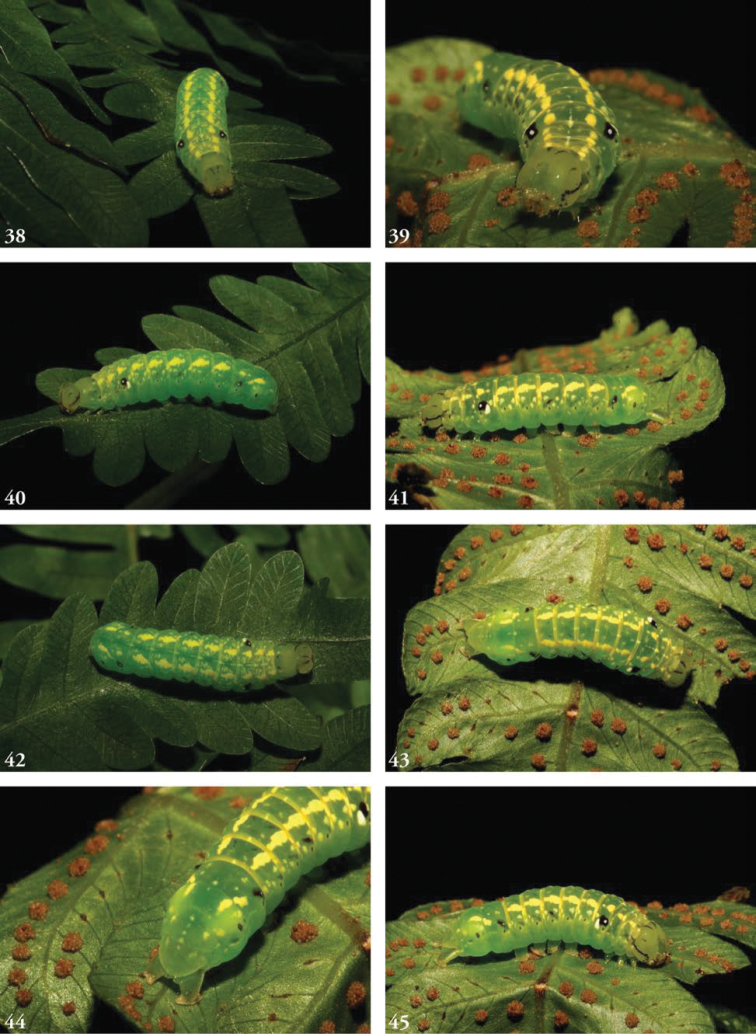
Larvae, green, penultimate instar. **39** 05-SRNP-48764 DHJ408129 **40**USNMENT01463798, 10-SRNP-44267 ♀ DHJ478607 **41** 05-SRNP-48764 DHJ408127 **42**USNMENT01463798, 10-SRNP-44267 ♀ DHJ478603 **43**USNMENT01463798, 10-SRNP-44267 ♀ DHJ478606 **44**USNMENT01463798, 10-SRNP-44267 ♀ DHJ478605 **45**USNMENT01463798, 10-SRNP-44267 ♀ DHJ478608 **46** 05-SRNP-48764 DHJ408128.

***Female genitalia***. As above, for genus.

##### Immature stages.

Known only from images of living larvae. Larvae are cylindrical, not tapered posteriorly, exhibiting green-brown polymorphism (in some cases the penultimate instar green, ultimate instar brown), and both bear false eyespots on the first abdominal segment; the spot is white postero-ventrally, the front half black with a white dot. In the brown form, the head bears a calico pattern, while in the green form the head is more uniformly green; both forms bear a lateral genal stripe. The brown form is predominantly rusty orange, a pair of subdorsal stripes formed by paler orange wedges. Larvae curl their heads under their abdomens when disturbed, emphasizing their false eyespots. This recall’s Yen and Wu’s (2009) remarks on the eriopine genus *Callopistria* which “when threatened...bend their abdominal segments A1 and A2 upwards,” as do caterpillars in many families. The subdorsal lines in the green larval form comprise a series of irregularly shaped segmental yellow blotches through A6, beneath which runs a series of supra-spiracular spots; a small supra-spiracular black dash may is visible on A4 and A7 as well. The posterior segments, beginning with A7, bear scattered yellow markings.

##### Biology.

Larvae collected in March, May, June, September, October, and November, with adults eclosing in each of the corresponding subsequent months. Recorded times from the onset of pre-pupa to eclosion 15–17 days. Pupation within a cocoon of leaves lightly silked together. Larvae have been collected feeding on the following ferns: *Pterisplumula* Desv. (= *Pterisquadriaurita* Retz., Pteridaceae), *Pteridiumcaudatum* (L.) (Dennstaedtiaceae), *Thelypterisnicaraguensis* (E. Fourn.) C.V. Morton (Thelypteridaceae), *Salpichlaenavolubilis* (Kauf.) J. Sm. (Blechnaceae), *Lomariopsisvestita* E. Fourn. (Lomariopsidaceae), and *Nephrolepisbiserrata* (Sw.) Schott (Davailleaceae). Recorded hymeopteran parasitoids at ACG include *Enicospilusmaculipennis* (Cameron, 1886) (Ichneumonidae: Ophioninae) and at least one undescribed ichneumonid (cocoon, Fig. 50). False eyespots on the first abdominal segment appear well developed by the third instar and conspicuous in the green fourth instar as well as the brown last instars. It may be no more than coincidence that all the brown last instars turned out to be males when reared, while all the green penultimate instars turned out to be females when reared.

**Figures 46–53.**
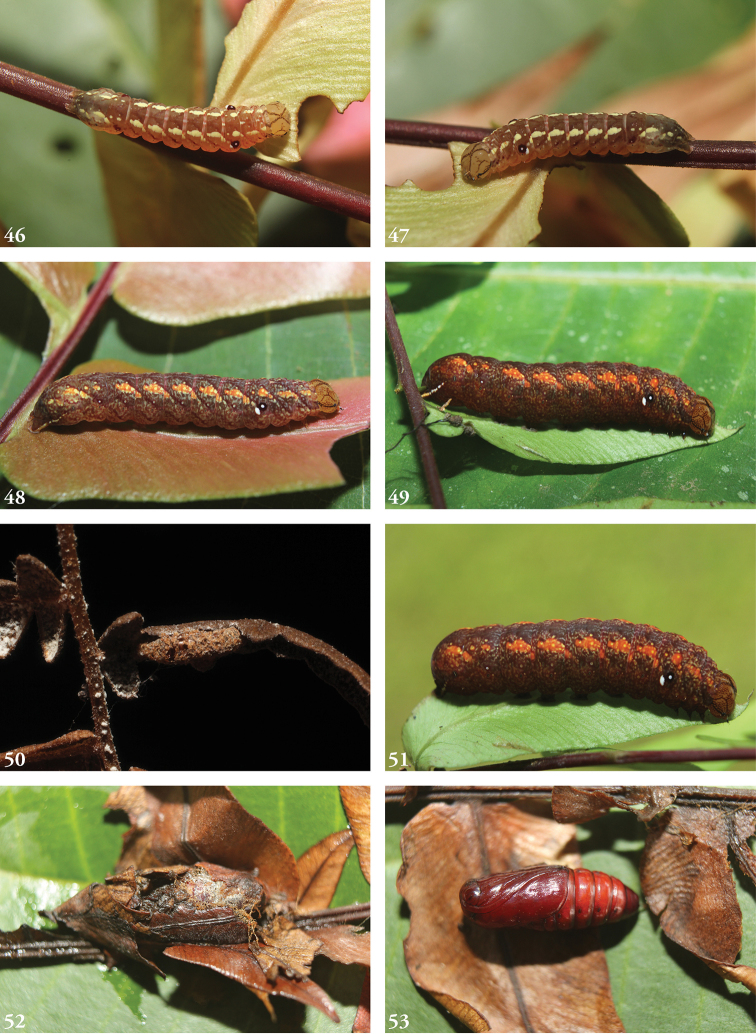
Young larvae, cocoon and pupa **46–49, 51–53** 14-SRNP-71583 **46** DHJ723850 **47** DHJ723852 **48** DHJ723855 **49** DHJ723892 **50** Ichneumonid cocoon, 11-SRNP-69437, DHJ804089 **51** DHJ723898 **52** DHJ723899 **53** DHJ723901.

##### Distribution.

Mid-elevation rainforest Mexico, Guatemala, Costa Rica.

## Discussion

Fern-feeding within the Noctuoidea is confined primarily to the Eriopinae, a small number of genera with uncertain placement, and several genera of Herminiinae (Erebidae). The degree to which fern-feeding is more generally conserved phylogenetically has yet to be rigorously tested but see Weintraub et al. (1995) for such an examination in the Lithinini (Geometridae). Within the Noctuidae*sensu stricto*, a precise determination of the number of fern-feeding origins is only now feasible, and enough phylogenetic information exists at least to imply its existence, if not its independent origin, outside the Eriopinae proper. Because of the uncertain placement of several noctuid genera, including *Leucosigma* (Goldstein et al. 2018a), *Lophomyra* (Goldstein et al. 2018b), and *Fagitana* Walker, the number of inferred origins of noctuid fern-feeding might increase with further study.

In addition to the exercise of circumscribing such genera as well as the Eriopinae, questions remain among the higher-level assignments of those with no obvious relatives, including *Aprica*. Although images of larvae reveal certain features common to many fern-feeding caterpillars (green-brown polymorphism, the presence of eyespots on A1, cephalic striping), most of the larval characters discussed by Beck (1960, 1999–2000), Poole (1995), Fibiger and Lafontaine (2005), and Yen and Wu (2009) that could potentially corroborate a higher taxonomic cannot be visualized from available habitus images of living larvae. Adult features considered diagnostic for Eriopinae (e.g., uniquely configured abdominal brushes, eversible saccular coremata, simple membranous corpus bursae without signa) are not observed in *Apricapatula*. Although fern-feeding is phylogenetically localized enough to have flagged this species for examination initially and cast further doubt on its placement in Bagisarinae or Xylenini, it should be noted that Ferguson (1997) removed it from *Bagisara* without the benefit of any life history information, and we detect insufficient evidence among the available data to place it with Eriopinae.

Provisional DNA barcode analyses suggest a possible kinship of *Aprica* with *Fagitana*, but these data are not adequate to corroborate their kinship in the absence of other characters, particularly among the larvae. It warrants mention in part because, like *Aprica*, the phylogenetic placement of *Fagitana* is uncertain; it is a ditypic genus comprising the well-characterized North American species *littera* associated with ferns in at least three of the same families as hosts of *Aprica* (Blechnaceae, Thelypteridaceae, Dennstaedtiaceae) and a rather dissimilar Asian species *gigantea* (Draudt, 1950) with an unknown life history. While *Aprica* and *Fagitana* share the unusual larval fern-feeding behavior with known Eriopinae, this is insufficient to unite them given the absence of published diagnostic eriopine genitalic features. Although we find it less than ideal to have created a monotypic genus, *Aprica*, these discrepancies combined with the absence of larval characters and more extensive phylogenetic data render its placement elsewhere difficult to support, and its higher placement at best ambiguous.

## Supplementary Material

XML Treatment for Aprica

XML Treatment for Aprica patula
